# *Pseudomonas aeruginosa* utilizes the host-derived polyamine spermidine to facilitate antimicrobial tolerance

**DOI:** 10.1172/jci.insight.158879

**Published:** 2022-11-22

**Authors:** Chowdhury M. Hasan, Sian Pottenger, Angharad E. Green, Adrienne A. Cox, Jack S. White, Trevor Jones, Craig Winstanley, Aras Kadioglu, Megan H. Wright, Daniel R. Neill, Joanne L. Fothergill

**Affiliations:** 1 Department of Clinical Infection, Microbiology and Immunology, Institute of Infection, Veterinary and Ecological Sciences, University of Liverpool, Liverpool, United Kingdom.; 2 School of Chemistry and Astbury Centre for Structural Molecular Biology, University of Leeds, Leeds, United Kingdom.

**Keywords:** Infectious disease, Microbiology, Bacterial infections, Drug therapy, Polyamines

## Abstract

*Pseudomonas aeruginosa* undergoes diversification during infection of the cystic fibrosis (CF) lung. Understanding these changes requires model systems that capture the complexity of the CF lung environment. We previously identified loss-of-function mutations in the 2-component regulatory system sensor kinase gene *pmrB* in *P. aeruginosa* from CF lung infections and from experimental infection of mice. Here, we demonstrate that, while such mutations lowered in vitro minimum inhibitory concentrations for multiple antimicrobial classes, this was not reflected in increased antibiotic susceptibility in vivo. Loss of PmrB impaired aminoarabinose modification of LPS, increasing the negative charge of the outer membrane and promoting uptake of cationic antimicrobials. However, in vivo, this could be offset by increased membrane binding of other positively charged molecules present in lungs. The polyamine spermidine readily coated the surface of PmrB*-*deficient *P. aeruginosa*, reducing susceptibility to antibiotics that rely on charge differences to bind the outer membrane and increasing biofilm formation. Spermidine was elevated in lungs during *P. aeruginosa* infection in mice and during episodes of antimicrobial treatment in people with CF. These findings highlight the need to study antimicrobial resistance under clinically relevant environmental conditions. Microbial mutations carrying fitness costs in vitro may be advantageous during infection, where host resources can be utilized.

## Graphical abstract

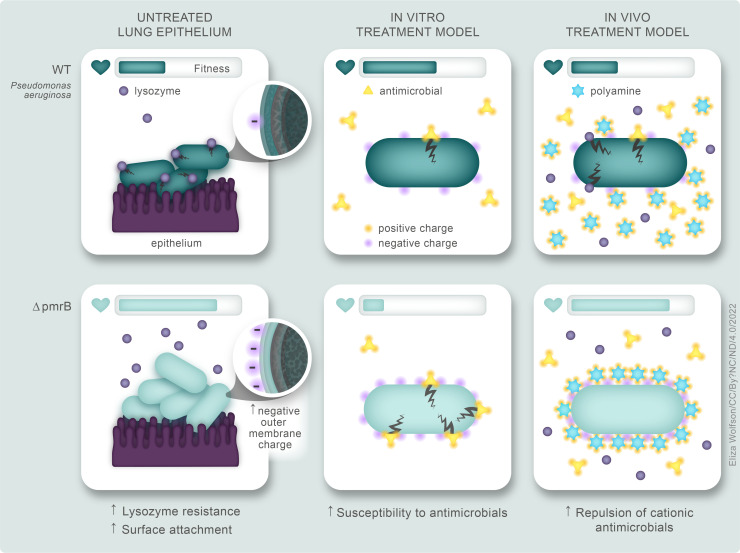

## Introduction

*Pseudomonas aeruginosa* is a ubiquitous environmental bacterium and a metabolically versatile opportunistic pathogen, responsible for severe, acute nosocomial infections (1, 2). It is also the most frequently recovered pathogen of the cystic fibrosis (CF) lung (3), where it causes chronic infection that is associated with pulmonary exacerbations and declining lung function. Such infections are difficult to treat, due in part to intrinsic antimicrobial resistance of *P*. *aeruginosa*, together with adaptive resistance mechanisms induced by the presence of antimicrobial agents or other environmental factors (4). The chronic nature of *P*. *aeruginosa* infection of the CF lung necessitates long-term, high-dose antimicrobial therapy, creating conditions conducive to the emergence and selection of acquired resistance mechanisms (5). Phenotypic flexibility and a large genome-encoding complex regulatory machinery makes chronically colonized *P*. *aeruginosa* a challenge to eradicate and necessitates frequent review of treatment regimens for people with CF.

LPS is a key component of the Gram-negative outer membrane; it can be stabilized by the addition of divalent cations, including Mg^2+^ and Ca^2+^. Cationic antimicrobials, including polymyxin B, colistin, and host-derived peptides, such as LL37, exert their effects via disruption of cell membrane integrity, but they rely on charge differentials with the outer membrane in order to bind (6). Modifications of LPS that reduce cationic antimicrobial binding affinity and penetration can result in resistance (7). One such modification is the addition of positively charged 4-amino-4-deoxy-L-arabinose to the lipid A component of LPS (8), mediated in *P*. *aeruginosa* by the proteins encoded by the *arnBCADTEF-ugd* operon (9). Expression of operon genes is regulated by 2-component signaling systems, such as PhoPQ and PmrAB (10, 11), which are activated under conditions of locally decreased divalent cation concentrations. This ensures that the charge of the membrane can be maintained when Mg^2+^ and Ca^2+^ are limited. PhoPQ- and PmrAB-induced expression of the *arn* operon results in high level resistance to both cationic peptides and aminoglycosides (12). Specific mutations in *pmrAB* have been implicated in polymyxin resistance via upregulation of both the lipid A deacylase *pagL* and the *arn* operon (13–15).

Expression of genes *PA4775*, *speE2* (*PA4774*), and *speD2* (*PA4773)* (16)*,* located adjacent to *pmrAB* on the chromosome*,* is also induced by Mg^2+^-limiting conditions, due to the presence of a PmrA binding site close to *speD2* (6). These genes (PA4773-5) are involved in the synthesis of the polyamine spermidine. During environmental stress or periods of low cation availability, PmrAB stimulates polyamine synthesis and these polyamines coat the bacterial surface, increasing the outer membrane charge and providing protection against both antimicrobial agents and oxidative stress (6).

Polyamines are polycationic hydrocarbons, containing 2 or more amine groups; they are abundant across all 3 kingdoms of life. Polyamine utilization and uptake genes are found in all bacteria (17) and species, including *P*. *aeruginosa*, can utilize polyamines as a sole carbon source for growth (18). Putrescine, spermidine, and cadaverine, the principal polyamines of prokaryotes, have been implicated in iron and free radical scavenging, acid resistance, biofilm formation, protection from the phagolysosome, interaction with components of cell envelopes, and antimicrobial resistance (17). However, there is uncertainty regarding the effect, if any, that polyamines have on antimicrobial susceptibility in *P*. *aeruginosa*. Increased susceptibility to multiple classes of antibiotics was observed when PAO1 was cultured with polyamines, (19) but addition of exogenous polyamines to a PAO1 lacking a functional spermidine synthase (*speE2*) partially protected the outer membrane from polymyxin B (6). While the extent of the role played by polyamines in *P*. *aeruginosa* growth, virulence, and antimicrobial resistance has not been fully determined, it is notable that spermine was found to be elevated in the airways of those with CF and that levels have been reported to decrease during treatment of pulmonary exacerbations (20), while those of putrescine have been found to decrease (21).

In a previous study, we identified loss-of-function mutations in *pmrB* in *P*. *aeruginosa* isolated from the airways of mice, following experimental infection, and in isolates taken from people with CF (22, 23). These isolates showed enhanced susceptibility to multiple classes of antibiotics. Here, we sought to understand why loss-of-function *pmrB* mutations might be retained in *P*. *aeruginosa*, in an environment of prolonged antimicrobial exposure, such as the CF lung. As we had observed altered LPS structure in *pmrB* mutants (23), we hypothesized that host-derived molecules might play a role in stabilizing the outer membrane of *P*. *aeruginosa* in vivo, thereby overcoming the lack of PmrAB-driven modifications of lipid A. Here, we propose that the host cationic polyamine spermidine acts in this way, negating the antimicrobial susceptibility phenotype of *P*. *aeruginosa*, which lacks functional PmrB. These findings highlight the need to conduct antimicrobial susceptibility testing under environmentally relevant conditions.

## Results

### P. aeruginosa lacking PmrB show enhanced antimicrobial susceptibility in vitro but not in vivo.

**Figure 1:**
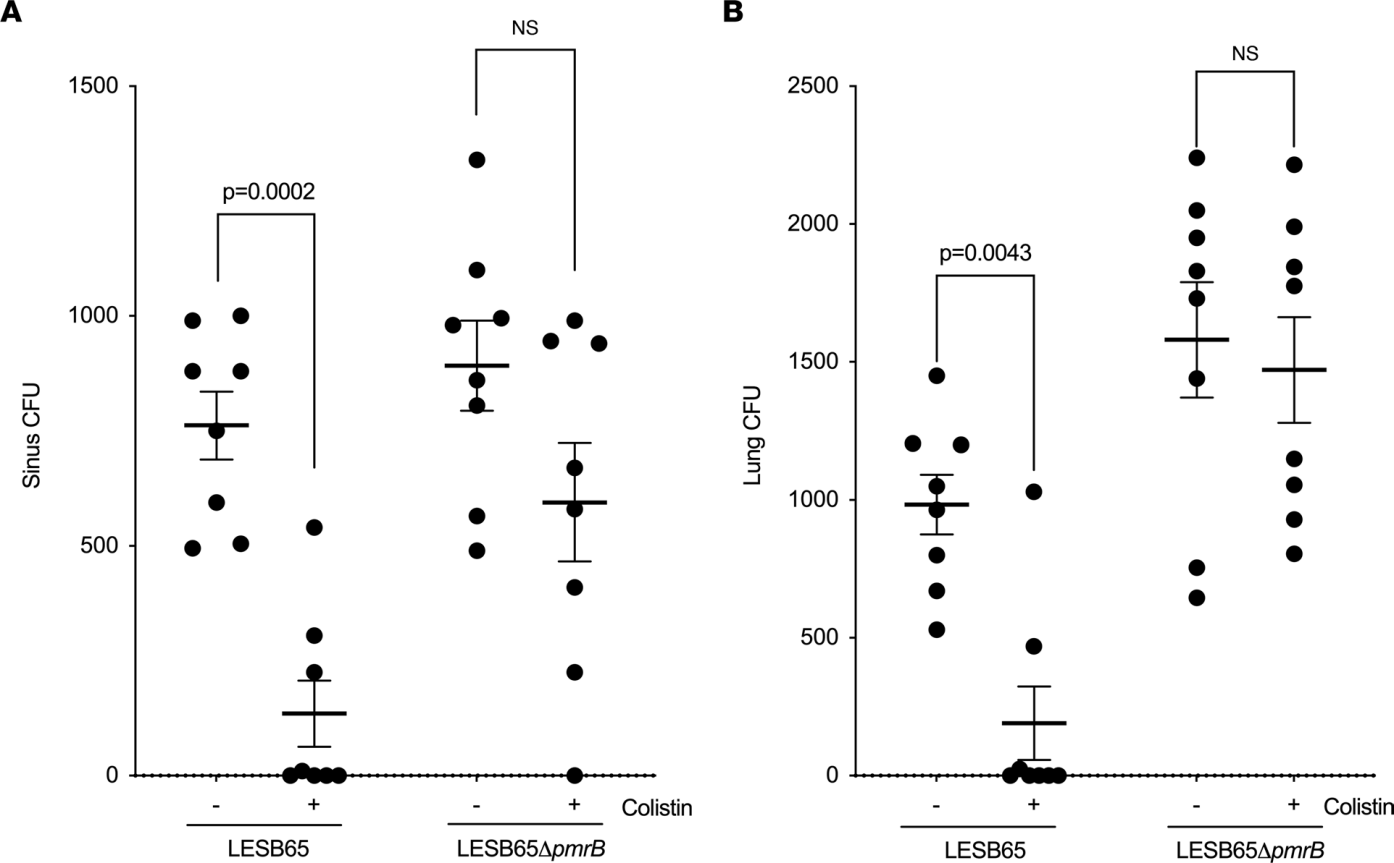
Difference in in vitro and in vivo antimicrobial susceptibility in PmrB-deficient *P*. ***aeruginosa*****.** LESB65 or LESB65Δ*pmrB* CFU in (**A**) sinuses and (**B**) lungs at 48 hours after infection. Mice were intranasally infected with 2 × 10^6^ CFU *P*. *aeruginosa*, and at 6 and 24 hours after infection, mice were intranasally administered a 50 μL dose of 20 μg colistin or PBS control. Each circle represents an individual mouse, and *P* values were determined by 2-way ANOVA with Bonferroni’s correction. Data are representative of 2 independent experiments; *n* = 8 for each treatment group. Data are shown as mean ± SD

We previously described susceptibility to multiple classes of antibiotics in *P*. *aeruginosa* with naturally acquired loss-of-function mutations in *pmrB* and in a *pmrB*-deletion strain of LESB65 (23). To determine whether this susceptibility would result in improved infection outcomes following onset of antimicrobial therapy, we infected mice with LESB65 or a LESB65 mutant lacking *pmrB* (LESB65Δ*pmrB*) and then treated them with intranasal colistin at 6 and 24 hours after infection. In LESB65-infected mice, colistin treatment led to significant reductions in the number of *P*. *aeruginosa* recovered from both the upper airways (nasopharynx and sinuses) (Figure 1A) and the lungs (Figure 1B), with 4 of 8 mice clearing the infection completely. By contrast, colistin treatment did not significantly alter the bacterial burdens recovered from LESB65Δ*pmrB*-infected animals (Figure 1). Consistent with our previous findings, the LESB65Δ*pmrB* strain colonized lungs at a higher density than its wild-type parent strain (Figure 1B). We subsequently performed in vitro antimicrobial susceptibility testing with bacteria recovered from the infections and confirmed that LESB65Δ*pmrB* retained its susceptibility to colistin in vitro (Supplemental Figure 1; supplemental material available online with this article; https://doi.org/10.1172/jci.insight.158879DS1).

### Proteomics analysis suggests a switch from polyamine synthesis to uptake and utilization in LESB65ΔpmrB.

**Figure 2:**
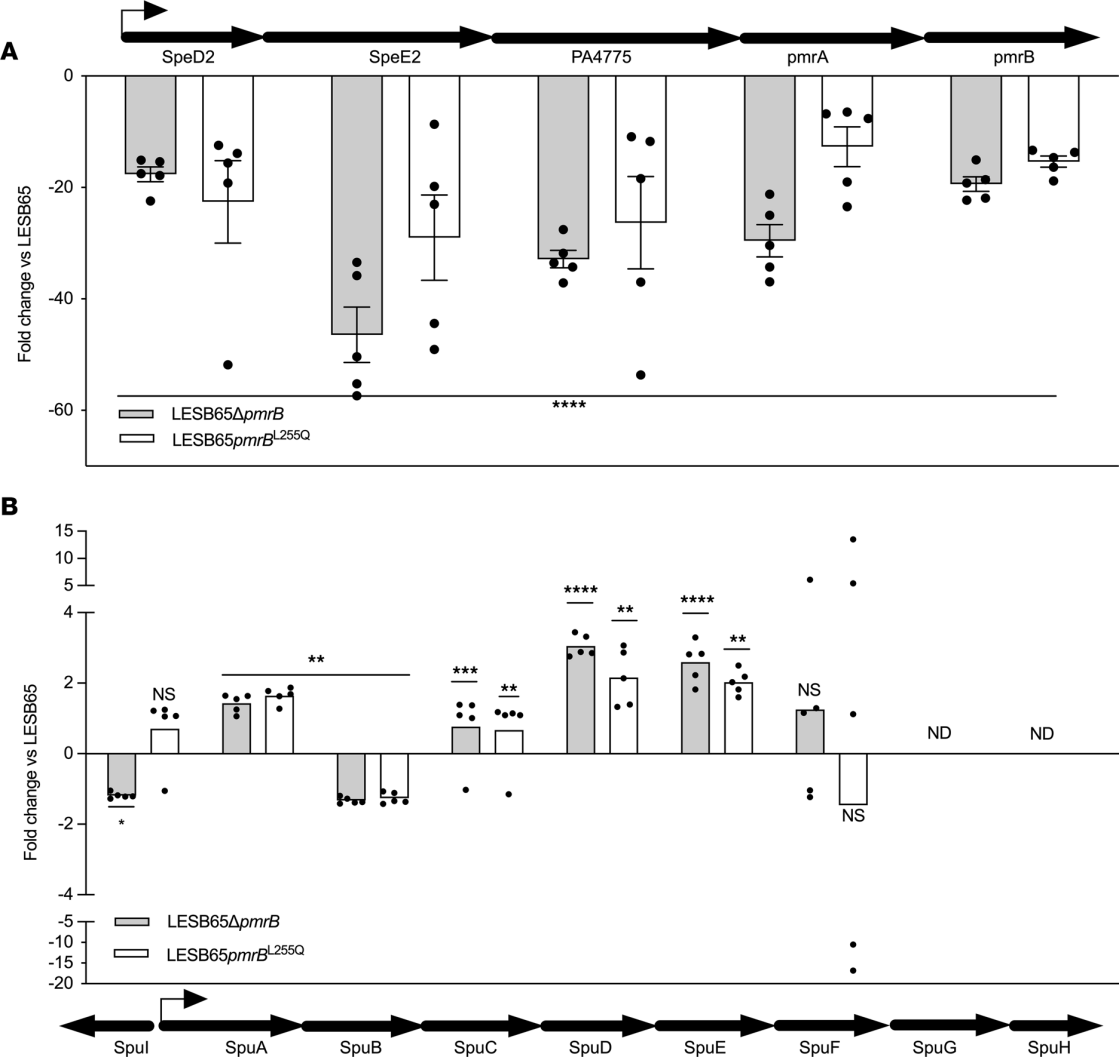
Loss of PmrB influences the relative abundance of polyamine synthesis and utilization proteins in *P*. ***aeruginosa*****.** Abundance of proteins of (**A**) the spermidine synthesis operon and (**B**) the polyamine binding, uptake, and utilization operon in LESB65Δ*pmrB* and LESB65*pmrB*^L255Q^ relative to LESB65. Values are fold change versus LESB65, and a negative value denotes decreased abundance. Data are shown as mean ± SEM (*n* = 5 per group), and significance was determined from label-free proteomics data using Progenesis QI. NS, adjusted *P* > 0.05; *P < 0.05; **P < 0.01; ***P < 0.001; ****P < 0.0001; ND, not detected. Statistical analysis (2-way ANOVA) of the data was performed using Progenesis QI for proteomics to identify significantly (*P* < 0.05, q ≤ 0.05, relative fold change ≥2) differentially expressed proteins. Block arrows denote gene position and orientation; line arrows show transcriptional start site and directionality. Source data can be found in Supplemental Data set 2 of Bricio-Moreno et al. (23).

To further explore the environment-dependent antimicrobial susceptibility profile of LESB65 and LESB65Δ*pmrB*, we revisited proteomics data obtained from late-exponential bacterial cultures grown in LB (23). We identified interstrain abundance differences in a group of functionally related proteins involved in polyamine transport, biosynthesis, and metabolism. Bacteria can synthesize polyamines or acquire them via environmental uptake. Genes involved in polyamine synthesis are cotranscribed with *pmrA* and *pmrB* (24), and the proteins encoded by those genes (SpeD2, SpeE2, and PA4775) were found at greatly reduced abundance both in LESB65Δ*pmrB* and in an LESB65-derived isolate with a naturally acquired missense mutation in *pmrB* (LESB65*pmrB*^L255Q^) (Figure 2A). There is a *pmrA* binding sequence close to the start codon of *speD2* (6), and these data suggest that, in the absence of a functional PmrAB system, expression of the operon is significantly (*P* < 0.0001) reduced. However, the reduced abundance of polyamine synthesis proteins in these strains appears to be offset by a corresponding increased abundance of the polyamine binding, uptake, and utilization proteins of the SpuABCDEFGH operon (Figure 2B). Of the 6 proteins of this operon that were detected in proteomics analysis, 4 were significantly more abundant in both LESB65Δ*pmrB* and LESB65*pmrB*^L255Q^, as compared with LESB65.

### Spermidine is abundant in both the airways and increases during infection and antimicrobial treatment.

**Figure 3:**
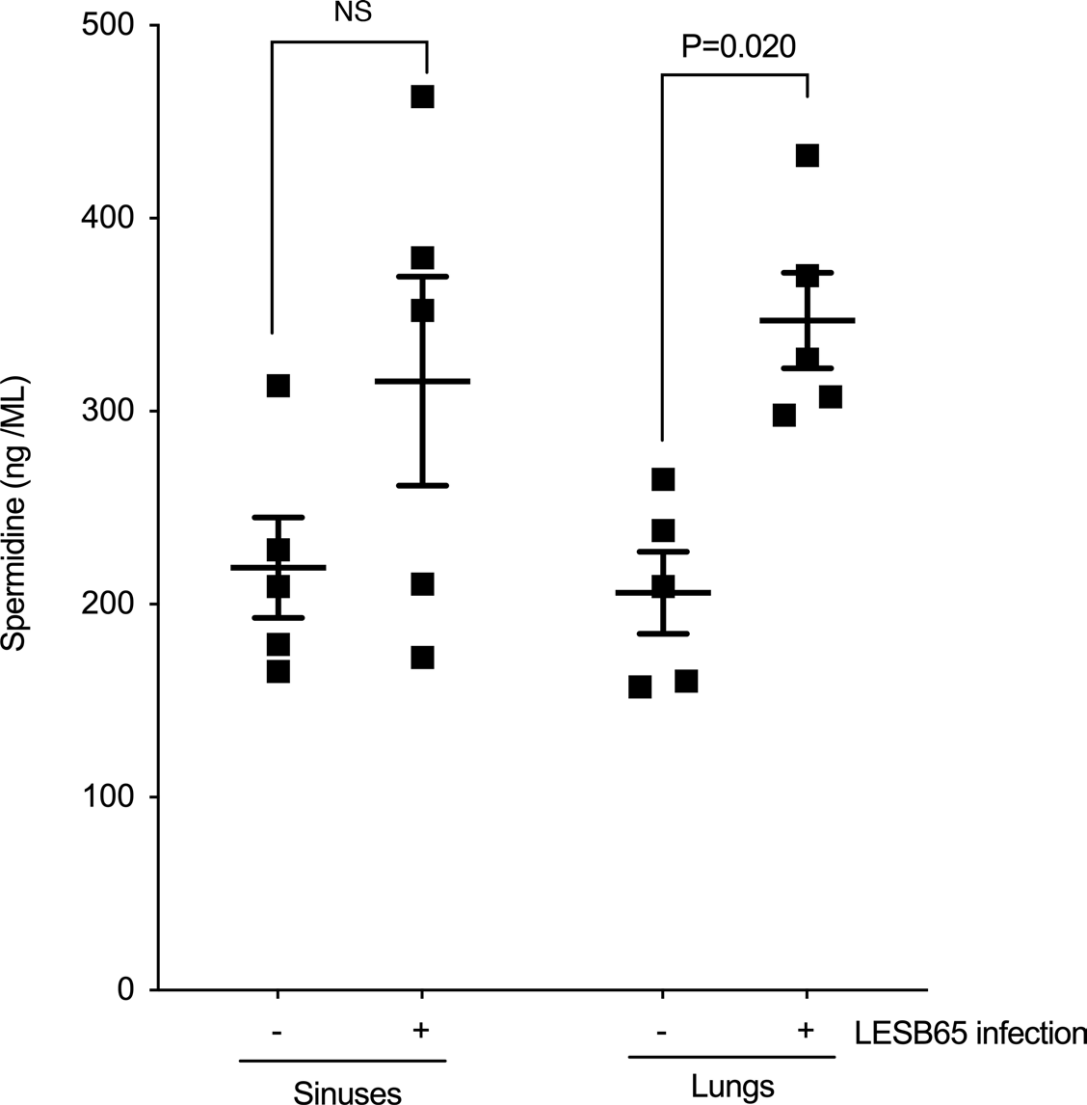
Spermidine is abundant in the murine respiratory tract, and bioavailability increases during *P*. ***aeruginosa*****infection.** Concentration of spermidine in the sinuses and lungs of mice at 48 hours after intranasal administration of PBS (–) or LESB65 (+). Spermidine was measured by ELISA, and each square represents a tissue sample from an individual mouse. Significance was determined by 2-way ANOVA with Bonferroni’s correction. Data are from a single experiment; *n* = 5 for each treatment group. Data are shown as mean ± SD.

The apparent increase in polyamine binding and acquisition proteins in the *pmrB* mutant strains may be advantageous in environments that are rich in free polyamines. Others have reported polyamine abundance in CF sputum and changes in bioavailability associated with pulmonary exacerbations (20, 21). As the spermidine synthesis proteins were significantly decreased in abundance in PmrB-deficient *P*. *aeruginosa*, we sought to determine whether the polyamine could instead be scavenged from the environment. We measured spermidine levels in the sinuses and lungs of both uninfected mice and those with chronic *P*. *aeruginosa* LESB65 infection (Figure 3). Spermidine was detectable at comparable concentrations in sinuses and lungs and was found to increase in the context of infection. This increase is unlikely to result from polyamine production in *P*. *aeruginosa*, as the levels of spermidine produced by high density cultures of LESB65 or LESB65Δ*pmrB* were approximately 1,000-fold lower than those detected in respiratory tissues (Supplemental Figure 2).

**Figure 4:**
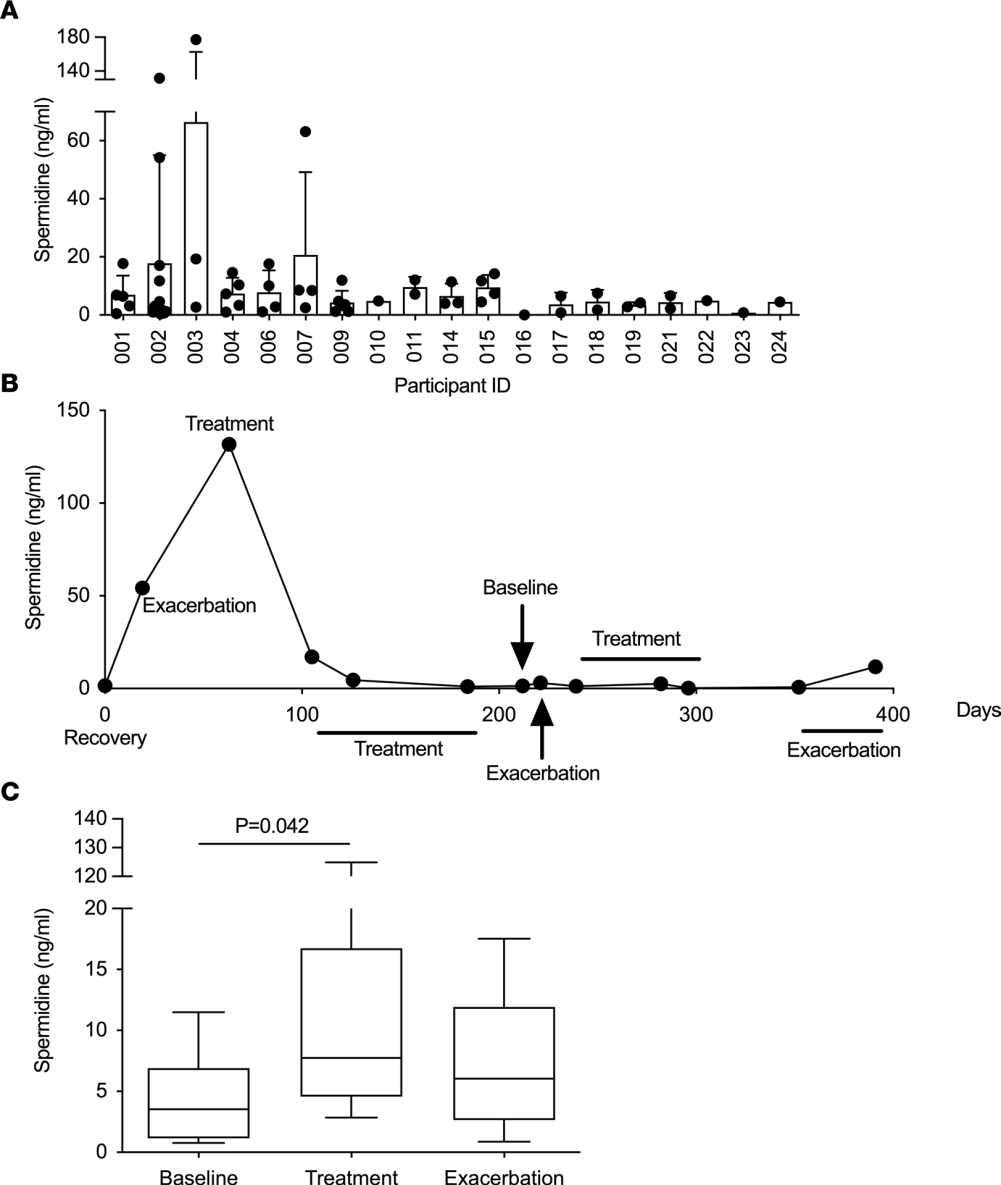
Spermidine is detectable in CF sputum. Concentration of spermidine in sputum from people with CF, chronically infected with *P*. *aeruginosa*, determined by competition ELISA. (**A**) Spermidine levels in 19 study participants. Between 1 and 13 samples were available per participant. No 2 samples from the same participant were collected at the same visit (*n* = 65). Data are shown as mean ± SD. (**B**) Changes in spermidine abundance in sputum from a single participant over time. This participant received meropenem during the treatment phases and ceftazidime during exacerbation and recovery/maintenance. The individual was coinfected with MRSA and so received teicoplanin throughout. (**C**) Collected samples were defined as baseline, treatment, or pulmonary exacerbation, defined by participant clinical data. Participants received 2 or more drugs, including aztreonam, fosfomycin, colistimethate, meropenem, tobramycin, colomycin, ceftazidime, teicoplanin and tazocin, with individual patient antibiotic combinations chosen by their specialist clinician. Whiskers show the 10–90 percentile. Significance was determined by 1-way ANOVA, with Dunnett’s multiple comparison testing. Data are from a single experiment.

We also detected free polyamines in sputum from people with CF with chronic *P*. *aeruginosa* infection (Figure 4). Spermidine was quantifiable in samples from 18 of 19 people tested (Figure 4A). There was considerable inter- (Figure 4A) and intraparticipant (Figure 4B) variability in sputum spermidine levels, but levels were higher during periods of antimicrobial treatment (Figure 4C), suggesting potential for polyamines to influence treatment efficacy.

Another polyamine, spermine, was detected in all sputum samples and was found to be more abundant, relative to spermidine. Like spermidine, there was considerable interparticipant variation in spermine levels (Supplemental Figure 3A); however, there were no significant differences among, exacerbation, and treatment samples (Supplemental Figure 3B). Spermine levels were also investigated in the lungs and sinuses of uninfected and *P*. *aeruginosa*–infected mice (Supplemental Figure 3C). Spermine levels were below the detection limit of the ELISA in all lung samples tested and in 7 of 10 sinus samples. Thus, while both spermidine and spermine are produced in both human and mouse respiratory tissue, their relative abundance appears to be species specific.

### PmrB genotype influences P. aeruginosa surface interactions with spermidine.

**Figure 5:**
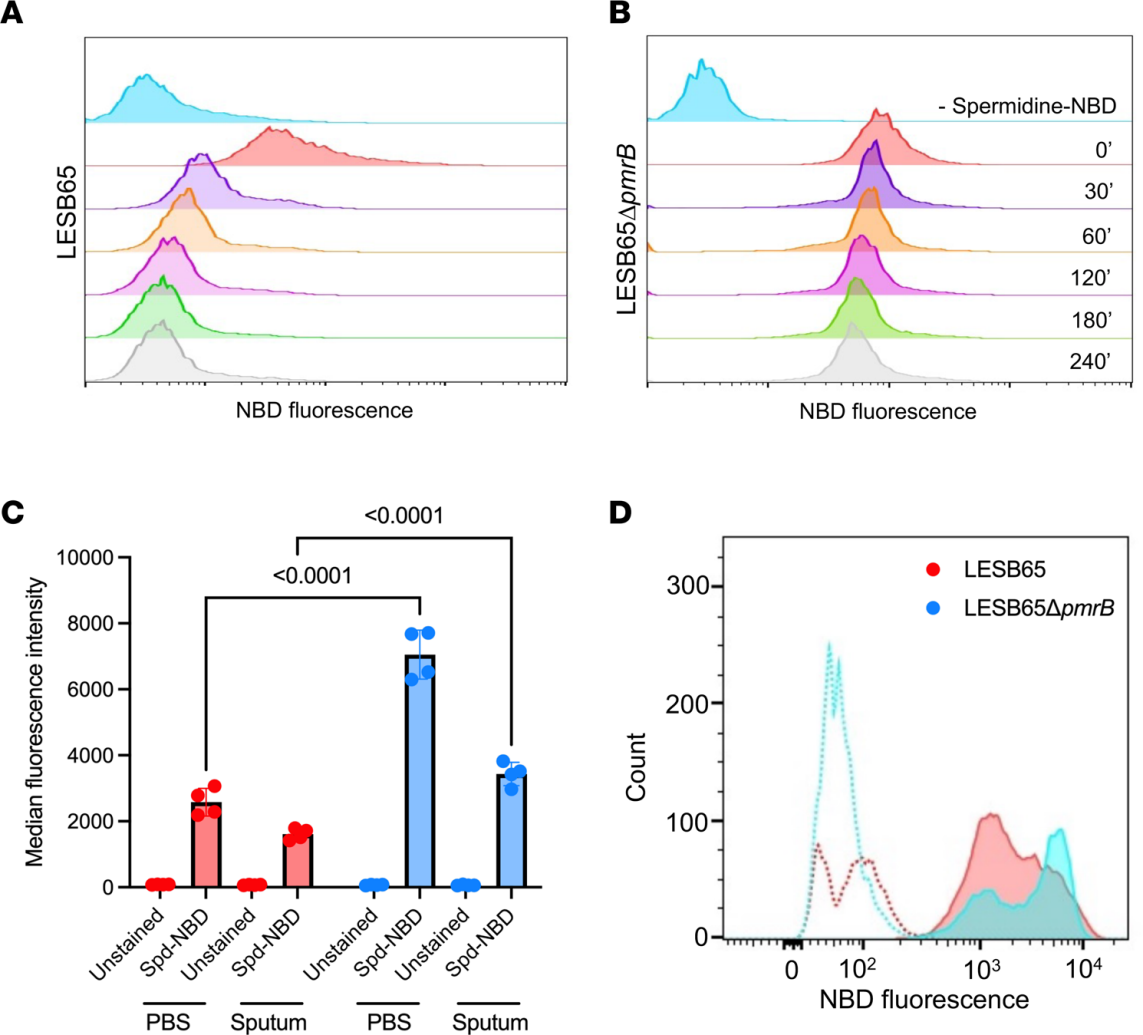
Prolonged interaction with environmental spermidine in PmrB-deficient *P*. ***aeruginosa*****.** (**A**) LESB65 and (**B**) LESB65Δ*pmrB* from mid-log cultures were incubated for 30 minutes with unlabeled spermidine (blue histogram) or spermidine-NBD (all other histograms), then pelleted, washed in saline, and resuspended in polyamine-free PBS. Spermidine-NBD binding to *P*. *aeruginosa* was determined by flow cytometry at 0, 30, 60, 120, 180 and 240 minutes after coincubation. Data are representative of 2 independent experiments, with *n* = 5 samples per group. (**C**) Spermidine was coincubated with LESB65 (red bars) or LESB65Δ*pmrB* (blue bars) for 30 minutes in PBS or in CF sputum. Bacteria were the pelleted, washed in saline and resuspended in PBS or CF sputum. Fluorescence was determined after 30 minutes by flow cytometry. Significance was determined by 2-way ANOVA with Šidák’s multiple comparison test. Data are representative of 2 independent experiments, with *n* = 4 samples per group. Data are shown as mean ± SD. (**D**) Representative flow cytometry histograms of LESB65 and LESB65Δ*pmrB* following 30 minutes in the presence (solid lines) or absence (dashed lines) of spermidine-NBD.

Having demonstrated that spermidine is available within the airways, we next sought to characterize whether *P*. *aeruginosa* might interact with this cationic molecule. Using purified spermidine tagged with fluorescent nitrobenzoxadiazole (NBD), we first investigated whether the polyamine could interact with the bacterial surface and if such interactions were transient or prolonged. For these assays, we used nontoxic concentrations of spermidine, determined by broth microdilution (Supplemental Figure 4). LESB65 and LESB65Δ*pmrB* were coincubated with 4 mM unlabeled spermidine or spermidine-NBD for 30 minutes before the bacteria were pelleted by centrifugation and resuspended in PBS. We then determined the extent of spermidine binding to the bacterial surface by flow cytometry. Spermidine-NBD bound both LESB65 and LESB65Δ*pmrB*, as evidenced by increasing median fluorescence intensity (MFI) for *P*. *aeruginosa* cocultured with labeled versus unlabeled spermidine (MFI, 10.4 vs. 3.00 for LESB65 with spermidine-NBD vs. unlabeled spermidine; MFI, 73.3 vs. 3.13 for LESB65Δ*pmrB*; *P* *<* 0.0001 for both strains) (Figure 5, A and B). The NBD fluorescence of the LESB65Δ*pmrB* population was significantly higher than that of LESB65 population (MFI, 73.3 LESB65Δ*pmrB* vs. 10.4 LESB65; *P* < 0.001). Furthermore, fluorescence was retained longer in LESB65Δ*pmrB* cultures (MFI, 73.3 at 0 minutes, 67.5 at 30 minutes, 62.3 at 60 minutes, 56.9 at 120 minutes, and 54.6 at 240 minutes) than in LESB65 cultures (MFI, 10.4 at 0 minutes, 7.30 at 30 minutes, 5.49 at 60 minutes, 4.60 at 120 minutes, and 4.59 at 240 minutes) (Figure 5, A and B), suggesting prolonged binding or uptake in the LESB65Δ*pmrB* strain. Binding to and surface coating of NBD-spermidine on the *P*. *aeruginosa* membrane was confirmed by fluorescence microscopy (Supplemental Figure 5).

We next investigated whether the same interactions between *P*. *aeruginosa* and spermidine might take place in more chemically complex environments, more reflective of CF lung conditions. To this end, we repeated the flow cytometry assay using irradiated CF sputum as the assay buffer. Binding to spermidine was reduced for both LESB65 and LESB65Δ*pmrB* in CF sputum, as compared with in PBS, although significant interaction was still apparent for both strains (Figure 5C). Furthermore, as was observed in PBS, LESB65Δ*pmrB* demonstrated a significantly enhanced spermidine binding capacity in CF sputum, relative to that of the wild-type control (Figure 5C). The staining pattern of both strains was altered in CF sputum, with evidence of brightly and dimly stained subpopulations of bacteria (Figure 5D). These subpopulations may result from dynamic modification of surface charge in response to PmrAB-independent environmental-sensing mechanisms activated in CF sputum.

### Surface-coated spermidine protects P. aeruginosa from antimicrobials and offsets the susceptibility associated with loss of PmrB function.

Polyamines have been implicated in resistance to several classes of antibiotics, with binding of these positively charged molecules to the *P*. *aeruginosa* membrane reducing charge interactions with cationic antimicrobials (6, 24). The decreased abundance of SpeD2, SpeE2, and PA4775 in loss-of-function *pmrB* mutants may, therefore, contribute to the observed increases in antimicrobial susceptibility, under conditions in which polyamines cannot be readily scavenged from the environment. However, where polyamines are abundant, the increased polyamine-binding potential of LESB65Δ*pmrB* might offset the inherent susceptibility to antimicrobials associated with loss of PmrB function. To explore this idea, we performed minimum inhibitory concentration (MIC) assays with *P*. *aeruginosa* without spermidine, in the presence of spermidine, or with *P*. *aeruginosa* that had been preincubated with spermidine and then pelleted and washed before addition of antibiotics (Table 1). Assays were performed with PmrB-deficient strains on both the LESB65 and PAO1 backgrounds. In both cases, spermidine increased the colistin MIC50 of the *pmrB*-deficient strain, but not the wild-type ancestor, by 4- to 8-fold. This was the case both when the spermidine was present throughout the assay and when strains were preincubated with the polyamine. In polyamine-rich environments, such as the respiratory tract, surface coating of *P*. *aeruginosa* with cationic polyamines may achieve a comparable outcome to PmrAB-driven L-Ara-4N addition to LPS lipid A by increasing the positive charge of the outer membrane. This finding may explain why loss-of-function *pmrB* mutations are retained in *P*. *aeruginosa*, causing chronic infection of the CF lung, despite prolonged, high-dose antimicrobial treatment.

### Exogenous spermidine influences P. aeruginosa biofilm formation.

**Figure 6:**
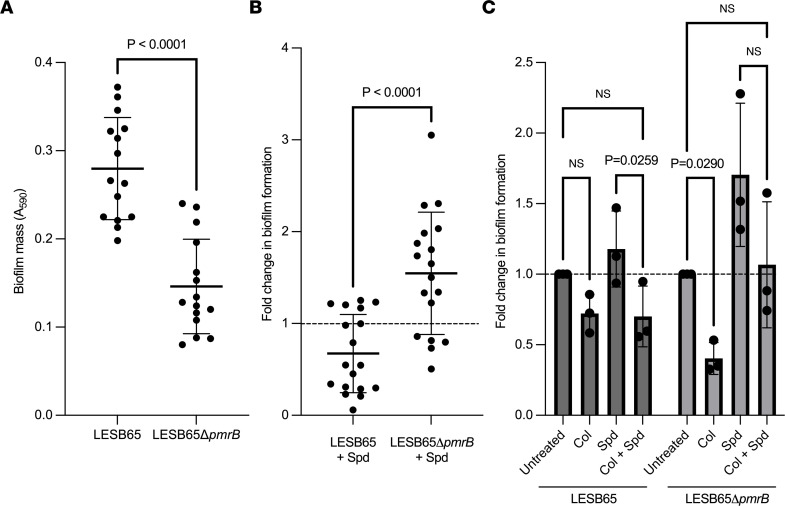
Spermidine promotes biofilm production in PmrB-deficient *P*. ***aeruginosa*****.** Surface-attached biofilm production was quantified by crystal violet staining. (**A**) Biofilm mass after 48 hours culture of LESB65 and LESB65Δ*pmrB*. (**B**) Fold change in biofilm production versus the no-spermidine control for LESB65 and LESB65Δ*pmrB*. (**C**) Biofilm formation, relative to untreated controls, in presence of 4 mM spermidine, 8 g/mL colistin, or both spermidine and colistin. Lines represent the mean, and data are shown as standard deviation. Data are shown as the mean ± SD. Data are representative of (**A** and **B**) 4 or (**C**) 3 independent experiments; *n* = 15 (**A**) or *n* =18 (**B**) for each treatment group. In **C**, each data point represents a biological replicate that is the mean of 5 technical replicates. *P* values are from (**A** and **B**) 2-tailed paired *t* tests or (**C**) 2-way ANOVA with Tukey’s multiple comparison test.

Polyamines contribute to biofilm formation in both Gram-positive and Gram-negative bacterial species (25). They serve as structural components of the extracellular matrix but also stimulate signaling that can promote or inhibit biofilm formation (26, 27). However, the role of polyamines in *P*. *aeruginosa* biofilm formation is not well described. To determine whether spermidine-induced reductions in antimicrobial susceptibility in LESB65Δ*pmrB* might by augmented by enhanced biofilm formation, we quantified surface-attached biofilm by crystal violet staining. LESB65 formed biofilm more readily than LESB65Δ*pmrB* (Figure 6A). However, while the addition of spermidine to cultures led to a modest reduction in LESB65 biofilm mass, it induced a significant increase in LESB65Δ*pmrB* biofilm mass over a 48-hour period (Figure 6B). LESB65Δ*pmrB* biofilms were more susceptible to colistin treatment than those formed by LESB65, but this susceptibility was lost with the addition of spermidine to cultures (Figure 6C). Thus, spermidine can enhance biofilm formation and promote biofilm tolerance to colistin via a mechanism dependent on *pmrB* genotype.

## Discussion

Despite their ubiquity and essentiality across all kingdoms of life, understanding of polyamine biology is limited. *P*. *aeruginosa* can synthesize polyamines from methionine, arginine, or ornithine but also scavenge them from their environment. In the context of infection, spermidine levels have been reported to be reduced in lungs in idiopathic pulmonary fibrosis, relative to those in healthy controls (28), while levels are increased in chronic obstructive pulmonary disease, particularly in those who smoke (29). It has been suggested that spermidine has a synergistic effect when coadministered with β-lactams for treatment of MRSA keratitis (19) but that polyamines also limit host-derived antimicrobial killing (30) and enhance *S*. *aureus* growth and virulence in this context (31). The expression of *P*. *aeruginosa* polyamine synthesis, uptake, and utilization genes is under environmental control, including by the PmrAB 2-component regulatory system.

The PmrAB locus is present in pathogenic Gram-negative species, including *P*. *aeruginosa*, *Klebsiella pneumoniae*, *Acinetobacter baumanii*, *E. coli*, and *Salmonella enterica* (32). The PmrAB system plays a key role in environmental sensing, stimulating production of polyamines and modification of LPS to buffer the outer membrane charge in conditions of low divalent cation availability (14). Mutations in *pmrB* are frequently identified in *P*. *aeruginosa* infecting the CF lung (33), and both activating and loss-of-function mutations have been described previously (14, 23). This apparent dichotomy may reflect environmental differences between individuals or between niches within the host. Where divalent cations or other positively charged molecules, including polyamines, can be co-opted to buffer membrane charge, loss-of-function *pmrB* mutations may be retained due to the advantages they confer in terms of lysozyme resistance and enhanced adherence to host surfaces (23). However, under conditions in which cationic molecules are scarce, activating mutations in *pmrB* would likely confer a greater advantage, by promoting the LPS modifications that reduce binding of antimicrobials to the bacterial outer membrane. Of note, *P*. *aeruginosa* has the capacity to actively promote polyamine bioavailability during infection. Contact with host cells induces a characteristic gene expression pattern in *P*. *aeruginosa* that leads to spermidine production and localization to the cell surface. This process is dependent on flagellar-mediated motility, with nonmotile bacteria failing to produce spermidine upon host cell contact (34). However, in the present study, the observed effects do not appear to be flagella dependent, as our findings were comparable with *pmrB* deletion mutants generated in both LESB65 and PAO1. PAO1 carries a single polar flagellum, while LESB65 lacks one.

The differences in the kinetics of interaction with spermidine in wild-type and PmrB-deficient LESB65 suggest that surface charge may influence the capacity of *P*. *aeruginosa* to utilize exogenous polyamines. While it is clear that detection of divalent cations through PhoPQ and PmrAB can modulate polyamine synthesis and uptake pathways, as well as inducing surface charge modifications, it remains to be determined whether direct sensing of polyamine abundance can modulate those same processes. An ability to alter surface charge, and, thus, change the efficiency of polyamine binding, in response to the local availability of those molecules, would offer advantages in metabolic resource management.

Similarly, while the findings presented here demonstrate spermidine binding to the surface of *P*. *aeruginosa,* it is unclear whether the changes in antimicrobial susceptibility and biofilm formation that we observed are a direct result of that physical interaction or whether they are a consequence of polyamine-induced signaling. The increase in positive charge associated with spermidine coating of the outer membrane may be sufficient to explain the increased resistance to cationic antimicrobials such as colistin, and spermidine might act as a substrate for biofilm formation, encourage greater surface interactions via the change in membrane charge, or aid in chelation of negatively charged biofilm DNA. However, we can’t rule out further contributions from spermidine-induced signaling, and others have reported an effect of exogenous polyamines on bacterial pathogen gene expression, including in *P*. *aeruginosa* (35, 36).

The composition and charge of the outer membrane of Gram-negative bacteria are key determinants of antimicrobial resistance and major barriers to antibiotic uptake (37). The findings presented here go some way toward explaining the dichotomy of retention of loss-of-function *pmrB* mutations in *P*. *aeruginosa*, in the face of antimicrobial pressure. The susceptibility of PmrB-deficient LESB65 to antimicrobials in vitro did not translate to susceptibility within the lung environment, and this may be explained by buffering of the negatively charged outer membrane with host-derived cationic molecules, including spermidine, together with the phenotypic advantages in vivo, including lysozyme resistance and increased attachment to host surfaces, that derive from loss of PmrB (23). This highlights the need to perform antimicrobial susceptibility testing under conditions that are relevant to infection and that capture key environmental cues that are sensed by pathogens or with which they interact. This is a particular challenge for those interested in pathogens of the CF lung, given the complexity of that environment and the difficulty in replicating its physical, chemical, and microbiological features in the laboratory. However, substantial progress has been made in this area (38) through use of metabolic profiling of CF pathogens (39) and analytical methods that utilize next-generation sequencing data to aid in the benchmarking of new models that aim to replicate conditions of the CF lung (40). As models continue to be refined, consideration should be given to the inclusion of host-derived factors, including polyamines, that might influence pathogen membrane charge and antimicrobial susceptibility.

## Methods

### Bacteria and culture conditions.

*P*. *aeruginosa* Liverpool Epidemic Strain B65 (LESB65), LESB65Δ*pmrB,* PAO1, and PAO1Δ*pmrB* were used throughout (23). Deletion of *pmrB* in LESB65 and PAO1 was performed as part of a previous study (23). Bacterial stocks were stored at –80°C in 15% (v/v) glycerol. Prior to experiments, isolates were streaked onto Mueller Hinton (MH) agar, and then liquid cultures were prepared in MH broth, unless otherwise stated, from a single colony, and incubated at 37°C in a shaking incubator (180 rpm).

### Sputum samples.

Sputum samples were collected from people with CF at the Adult CF centre (Liverpool Heart and Chest Hospital, Liverpool, United Kingdom) during periods of stable infection and periods of exacerbation. Samples were expectorated between 2017 and 2020 and stored at –80°C within 2 hours of production. Exacerbation definitions were physician based, using a clear set of criteria (drop in FEV_1_ of generally 10%, increased sputum production and discoloration, increased temperature measured on more than one occasion (>38°C), increased cough and dyspnea, malaise, lethargy, fatigue, poor appetite, and poor exercise tolerance). Treatment was dual therapy intravenous antibiotics, prescribed by specialist physicians on an individual patient basis.

### Chemicals and reagents.

Antibiotics and spermidine were purchased from Sigma-Aldrich. Stock solutions were prepared using DEPC water and filtered through a 0.22 μm syringe filter.

### LESB65 infection of mice.

All infections were performed at the University of Liverpool. Female 6- to 8-week-old BALB/c mice (Charles River) were used for infection experiments and housed in individually ventilated cages. Mice were acclimatized for 1 week prior to infection. Mice were randomly assigned to an experimental group on arrival at the unit by staff with no role in study design. For infection, 2 × 10^6^ CFU of midexponential growth *P*. *aeruginosa* were instilled into the nares of mice that had been lightly anesthetized with a mixture of isoflurane and oxygen. At 6 and 24 hours after infection, mice were intranasally administered a 50 μL dose of 400 μg/mL colistin in PBS or else PBS only for control animals, under light anesthesia. Following this, cage labels were reversed to blind researchers to experimental groups. Mice were culled at 48 hours after infection, and upper airway (sinus and nasopharynx) tissue and lungs were removed postmortem and homogenized in 3 mL PBS using an IKA T10 handheld tissue homogenizer. Homogenates were serially dilution onto *Pseudomonas* selective agar (Oxoid) for enumeration of infectious burden. Following enumeration, researchers were unblinded. No animals were excluded from analysis.

### Spermidine and spermine ELISA.

Spermidine was quantified from lysates of overnight *P*. *aeruginosa* cultures, from mouse upper airway (sinus and nasopharynx) and lower airway (lung) tissue homogenates, and from CF sputum. Bacterial lysates were prepared by sonication. Overnight cultures were pelleted by centrifugation and resuspended in 1 mL PBS prior to sonication. Mouse tissues were dissected at 48 hours after intranasal administration of 2 × 10^6^ CFU of midlog phase LESB65 (infected group) or PBS (control group). Competitive ELISA for spermidine (Abbexa) or spermine (MyBiosource) detection was performed in precoated 96-well plates, according to the manufacturer’s instructions.

### MIC assays.

MIC assays were performed by broth microdilution. Isolates were first streaked onto fresh MH agar, and then a single colony from each plate was further grown overnight in 5 mL MH broth on an orbital shaker (180 rpm) at 37°C. A fresh dilution in MH broth was made by incubating 200 μL of the overnight culture in 5 mL MH media. A hundred microliters of this culture was incubated in 96-well plates with 100 μL of 1:2 serially diluted antibiotic in MH broth. After a 24-hour static incubation at 37°C, the OD_600_ was determined to assess bacterial growth.

### MIC assays with spermidine.

MIC assays were performed as above, with the addition of 4 mM spermidine to the assay throughout or using *P*. *aeruginosa* that had been preincubated with 4 mM spermidine. In the latter case, overnight cultures of *P*. *aeruginosa*, prepared as above, were pelleted by centrifugation and resuspended in 5 mL PBS containing 4 mM spermidine. Tubes were incubated at 37°C and 1 g (180 rpm) for 30 minutes, and then bacteria were again pelleted and resuspended in MH broth for use in MIC assays.

### Synthesis of fluorescent spermidine-NBD (NBD-N8-spermidine; 7-nitro-2,1,3-benzoxadiazol-4-N8-spermidine hydrogen chloride).

A solution of N^1^-N^4^-Bis-Boc-spermidine (50 mg, 0.29 mmol) in acetonitrile (2.0 mL) was added to 4-chloro-7-nitrobenzofurazan (58 mg, 0.29 mmol) and cesium carbonate (94 mg, 0.29 mmol) and heated to 80°C under reflux conditions for 40 minutes. The reaction mixture was concentrated in vacuo, purified through a silica plug (60 Å, 40 – 63 micron), and flushed with EtOAc to elute the boc-protected fluorophore. The compound was then dissolved in a solution of 4 M HCl in dioxane (5 mL) and stirred for 3 hours at room temperature, before removing the solvent in vacuo. The crude mixture was dissolved in DCM was and extracted with water. The aqueous layer was lyophilized, yielding NBD-N8-Spermidine as the hydrochloride salt (41), a red, amorphous solid. The overall yield, following coupling and boc deprotection, was 74%, after both the addition of the fluorophore and the subsequent removal of the boc protection group. All commercial reagents and solvents were used as received. ^1^H NMR and ^13^C NMR spectra were recorded using a Bruker 400 ultra shield spectrometer. All chemical shifts are expressed in parts per million downfield from tetramethylsilane. Peak splittings are noted as singlet (s), doublet (d), triplet (t), quartet (q), pentet (p) and multiple (m) and combinations of the stated J coupling constants are recorded to the nearest 0.5 Hz. High resolution ES+ mass spectra were obtained on a Bruker MaXis Impact mass spectrometer.

δ_H_ (500 MHz, D_2_O) 8.48 (1 H, d, J 9.0 Hz, Ar 6-H), 6.35 (1 H, d, J 9.0 Hz, Ar 5-H), 3.66 (2 H, broad s, 2-H2), 3.17 (4 H, m, 5- and 7-H2), 3.11 (2 H, appt t, J 7.9 Hz, 9-H2), 2.08 (2 H, apt tt, J 8, 2.4 Hz, 8-H2), 1.88 (4 H, m, 3 and 4-H2). δ_C_ (125 MHz, D_2_O) 147.2 (Ar 4-C), 145.0 (Ar 7a-C), 144.7 (Ar 3a-C), 139.8 (Ar 5-C), 100.6 (Ar 6-C), 48.0 (5-C), 45.1 (7-C), 43.6 (2-C), 37.2 (9-C), 25.3 (3-C), 24.4 (8-C), 23.8 (4-C). HRMS (ESI): m/z calculated for C_13_H_20_N_6_O_3_ [M+H]^+^ 309.1675, found 309.1673.

### Treatment of CF sputum for use in flow cytometry.

Prior to use in spermidine binding assays, CF sputum was treated as follows: 1 mL sputum was irradiated with ultra-violet light for 20 minutes before minimal dilution in polyamine-free PBS and vigorous vortexing, as described by Devereux et al. (42). Treated CF sputum was split into 2 mL aliquots and stored at –80°C until required.

### Flow cytometry analysis of spermidine–P. aeruginosa interactions.

*P*. *aeruginosa* were preincubated with 4 mM spermidine-NBD or unlabeled spermidine, following the same protocol as that used for MIC assays. Following incubation, bacteria were pelleted by centrifugation and resuspended in either PBS or CF sputum. Immediately, and at 30, 60, 120, 180, and 240 minutes, 200 μL samples were removed from the cultures and analyzed for NBD fluorescence on a FACSAria II flow cytometer (BD Biosciences). Twenty thousand individual bacteria were recorded. Side-scatter and forward-scatter limits for bacterial flow cytometry were predetermined using *P*. *aeruginosa* stained with the DNA dye thiazole orange (Sigma-Aldrich).

### Biofilm assay.

Biofilm experiments were set up using overnight cultures. Each culture was first diluted 1:100 in fresh broth and then added to 96 well plates and incubated for 48 hours at 37°C under static conditions. Plates were washed twice with PBS and stained with 200 μL of a 0.25% solution of crystal violet in water. After incubating at room temperature for 15 minutes, the plates were rinsed twice with water and allowed to dry for 24 hours. The stain was then dissolved in 1 mL of 25% acetic acid in water and incubated at room temperature for 2 minutes. Biofilm formation was quantified by measuring the optical density of this final solution at 590 nm. To test the effect of spermidine on biofilm formation in the presence of colistin, cultures were set up as follows: a 1:100 dilution of each strain was added to 96-well plates with half of the wells supplemented with 4 mM spermidine. Plates were then incubated for 24 hours. After 24 hours, 1:2 serially diluted suspensions of colistin were added to both spermidine and spermidine biofilms. Plates were incubated for a further 24 hours before being processed as above.

### Statistics.

Data analysis was carried out using GraphPad Prism v.8.02 and JMP version 14.0. Data were tested for normality. One-way or 2-way ANOVA was used for comparison between groups, and post hoc analysis included correction for multiple comparisons (as stated in the figure legends). Significance was determined from label-free proteomics data using Progenesis QI. A *P* value of less than 0.05 was considered significant.

### Study approval.

Ethical approval for collection of CF sputum was obtained from the North West Research Ethics Committee, England, United Kingdom, IRAS 216408, ethics reference no 17/NW/0091). Written informed consent was obtained from all study participants, prior to enrollment. Ethical approval for animal studies was obtained from the UK Home Office (project license PP2072053) and the University of Liverpool Animal Welfare Ethical Review Board.

## Author contributions

CW, AK, MW, DRN, and JLF designed the study, contributed resources and reagents, and supervised staff. CMH, SP, AEG, AAC, TJ, JW, and DRN performed experiments. CMH, SP, AEG, AAC, MW, DRN, and JLF analyzed data. DRN, CMH, and JLF wrote the manuscript, with input from all authors.

## Supplementary Material

Supplemental data

## Figures and Tables

**Table 1:**
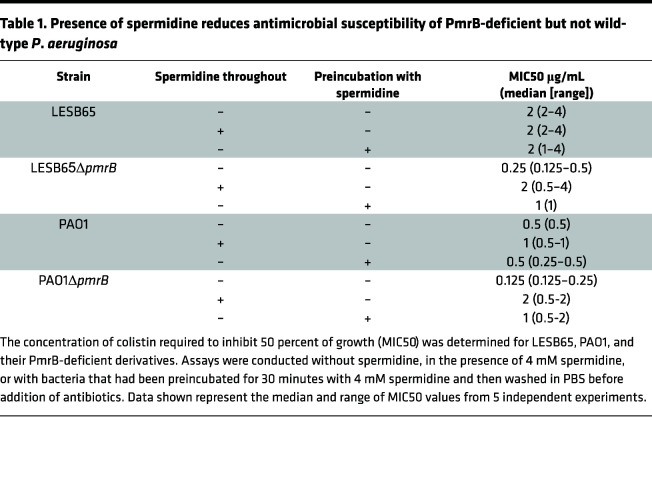
Presence of spermidine reduces antimicrobial susceptibility of PmrB-deficient but not wild-type *P*. *aeruginosa*

## Version 1 10/04/2022

In-Press Preview

## Version 2 11/22/2022

Electronic publication
