# Supplementary material for: Pseudomonas aeruginosa utilizes the host-derived polyamine spermidine to facilitate antimicrobial tolerance
Source: JCI Insight. 2022 Nov 22;7(22):e158879. doi: 10.1172/jci.insight.158879 (PMC9746822; doi:10.1172/jci.insight.158879)
Supplement: Supplemental data [file jciinsight-7-158879-s245.pdf]

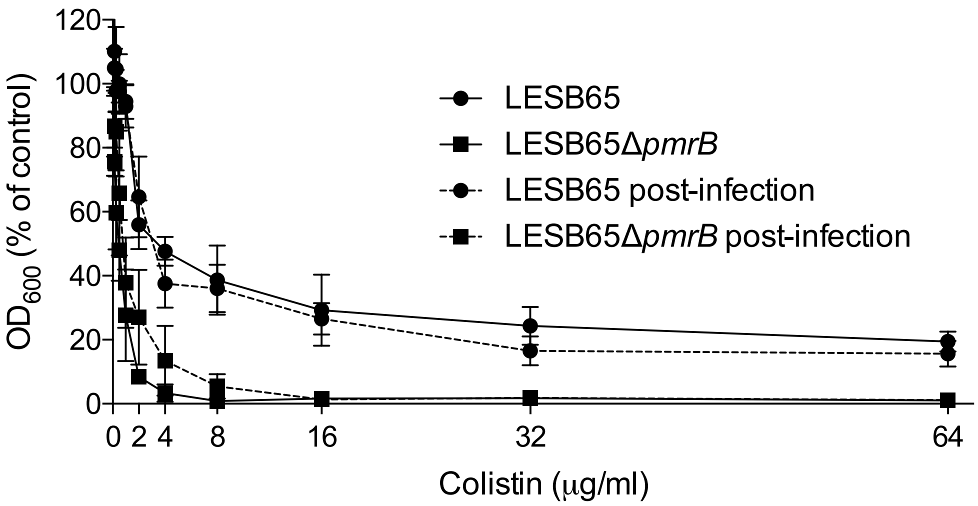

Supplementary Figure 1. Colistin sensitivity of LESB65 and LESB65Δ*pmrB* before and after two-day infection of mouse airways. Post-infection samples are *P. aeruginosa* recovered from 2 day post-infection mouse lung homogenates. Bacteria were recovered by plating homogenates onto Pseudomonas selective agar and then growth for ~30 hours at 37°C. Susceptibility testing was performed by microdilution in Mueller Hinton broth with two-fold decreasing concentrations of colistin (range 64-0.125μg/ml). Post-infection samples are from a single *in vivo* experiment. Frozen stocks of these bacteria were tested for susceptibility on three separate assay days. Data show mean OD_{600} ± standard deviation.

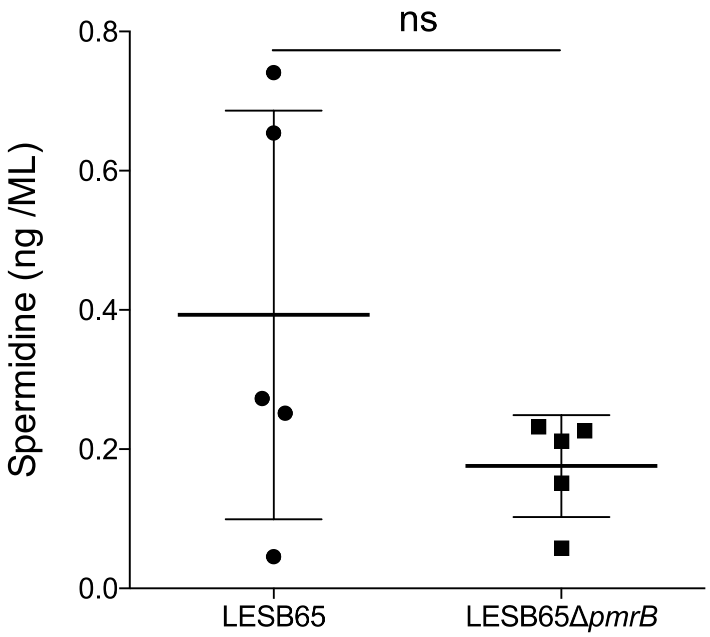

Supplementary Figure 2. Spermidine levels in bacterial lysates. Lysates of overnight cultures were used in a competitive spermidine ELISA. Levels detected were close to the manufacturer's stated limits of assay sensitivity (<0.28 ng/ml). Data are from five separate overnight cultures per strain, run, in duplicate, on a single ELISA assay plate. Ns = not significant.

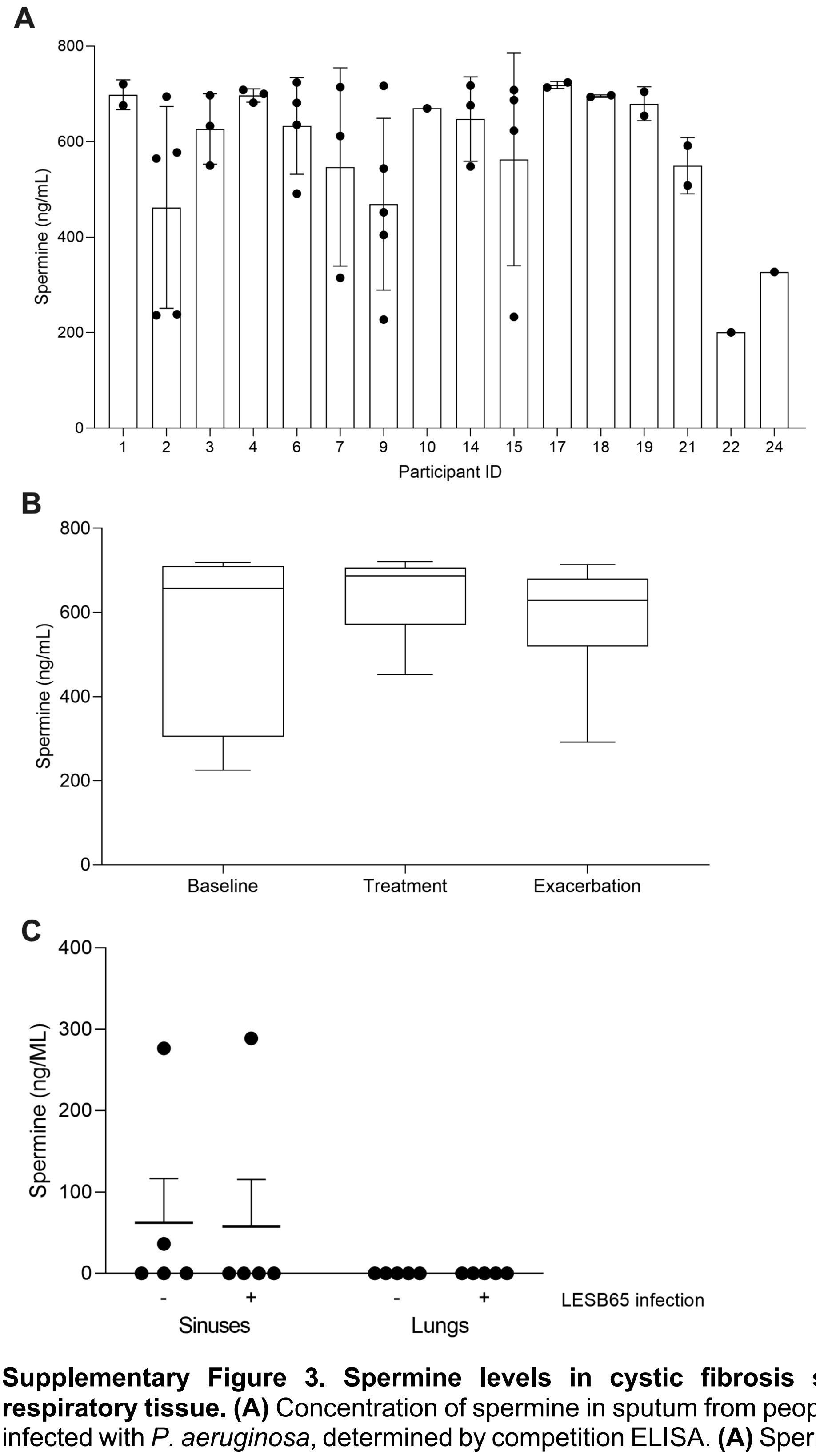

Supplementary Figure 3. Spermine levels in cystic fibrosis sputum and mouse respiratory tissue. (A) Concentration of spermine in sputum from people with CF, chronically infected with *P. aeruginosa*, determined by competition ELISA. **(A)** Spermine levels in 16 study participants. Between 1 and 5 samples were available per participant. No two samples from the same participant were collected at the same visit. **(B)** Collected samples were defined as baseline, treatment or pulmonary exacerbation, defined by participant clinical data. Whiskers show 10-90 percentile. **(C)** Concentration of spermine in the sinuses and lungs of mice at 48 hours post intranasal administration of PBS (-) or LESB65 (+). Each circle represents a tissue sample from an individual mouse. Data are from a single experiment.

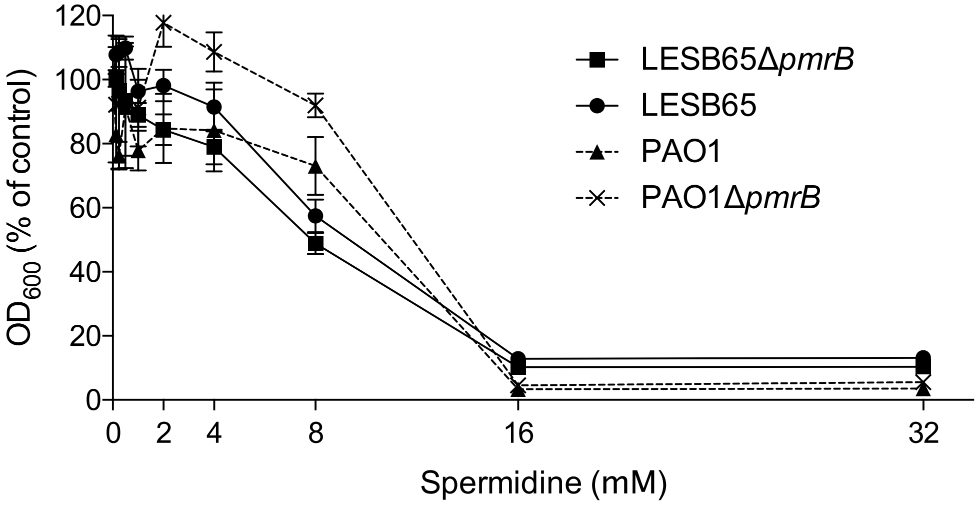

Supplementary Figure 4. Growth of *P. aeruginosa* in the presence of exogenous spermidine. LESB65, PAO1 and their isogenic *pmrB*-deficient mutants were grown in Mueller Hinton broth in the presence of spermidine (range 0.125-32 mM). Growth was determined after 24 hours by reading absorbance at 600 nM. A concentration of 4 mM was chosen for subsequent assays.

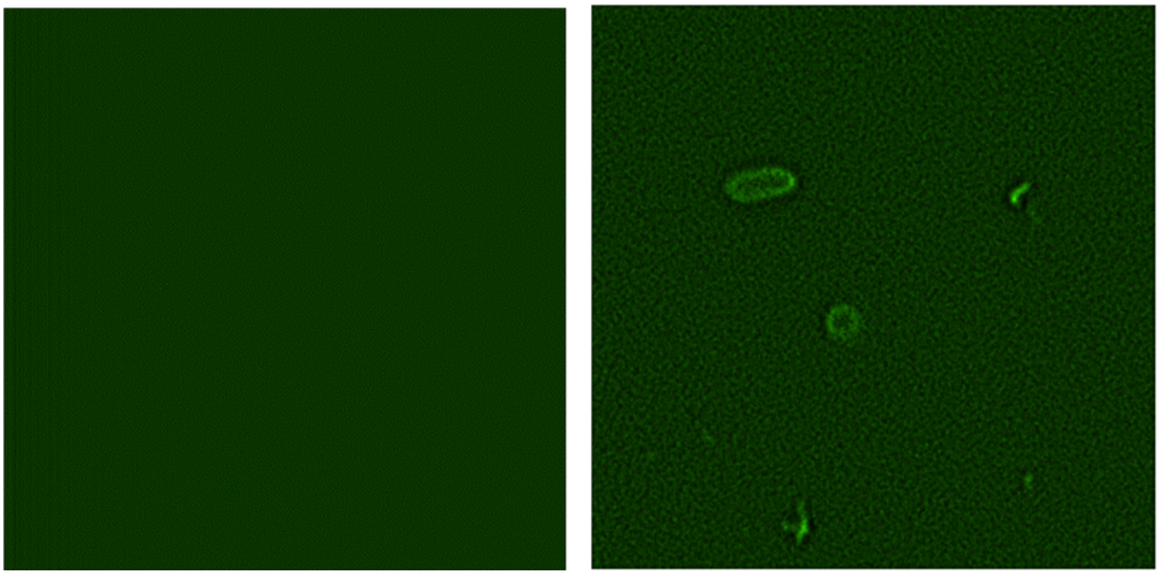

Supplementary Figure 5. Spermidine-NBD coats the *P. aeruginosa* surface. Fluorescence microscopy image of LESB65 in the absence (left) or presence (right) of spermidine-NBD.
